# Relationship between dyslipidemia and diabetic retinopathy in patients with type 2 diabetes mellitus: a systematic review and meta-analysis

**DOI:** 10.1186/s13643-023-02321-2

**Published:** 2023-08-24

**Authors:** Zhaoping Li, Yuan Yuan, Qianjin Qi, Qian Wang, Li Feng

**Affiliations:** 1 Department of Clinical Nutrition, Shandong Provincial Hospital Affiliated to Shandong First Medical University, 324 Jingwu Road, Jinan, 250021 Shandong China; 2 Shandong Provincial Hospital Affiliated to Shandong First Medical University, Jinan, 250021 Shandong China

**Keywords:** Diabetic retinopathy, Type 2 diabetes, Meta-analysis, Dyslipidemia

## Abstract

**Background:**

Diabetic retinopathy (DR) affects more than 80% of patients with diabetes. However, literature on the association between serum lipids and DR in patients with type 2 diabetes mellitus (T2DM) is inconsistent. Hence, in this study, we aimed to investigate the relationship between baseline serum lipids and the incidence of DR in patients with T2DM.

**Methods:**

We searched relevant articles in the PubMed, Embase databases, and the Cochrane Library up to February 7, 2022, and reviewed the reference lists of the included articles to identify appropriate cohort studies. The weighted mean difference (WMD) and the corresponding 95% confidence intervals (CIs) were calculated.

**Results:**

Thirteen cohort studies, including 7459 participants, were included in the present study. Higher levels of total cholesterol (2.94 mg/dL, 95% CI 1.32, 4.56), triglycerides (8.13 mg/dL, 95% CI 5.59, 10.66), and low-density lipoprotein cholesterol (2.53 mg/dL, 95% CI 1.02, 4.04) at baseline were observed in patients with later onset of DR. However, no significant difference in the high-density lipoprotein cholesterol level (0.27 mg/dL, 95% CI − 0.91, 1.45) was observed between patients with DR and without DR.

**Conclusion:**

The present results suggest that baseline triglyceride and cholesterol levels are significantly associated with the occurrence of DR in patients with T2DM. Thus, patients with T2DM may benefit from lowering serum lipids. Future studies exploring the relationship between longitudinal changes in serum lipids and DR occurrence are warranted.

**Systematic review registration:**

PROSPERO CRD42022319978

**Supplementary Information:**

The online version contains supplementary material available at 10.1186/s13643-023-02321-2.

## Introduction

The number of patients with type 2 diabetes mellitus (T2DM) is increasing yearly from 1990 onwards [1, 2]. As the previous literature reported [3], approximately 6.28% of the world’s population were affected by T2DM in 2017, and this prevalence is projected to increase to 7.08% by 2030 and 7.86% by 2040. T2DM has become a major public health problem worldwide.

Diabetic retinopathy (DR), one of the chronic microvascular complications of diabetes [4], affects more than 80% of patients with diabetes for whom the disease course exceeds 20 years and in some cases can lead to irreversible visual loss [5]. As the Vision Loss Expert Group reported [6], DR resulted a visual loss in 0.86 million patients who were above 50 years of age and became the fifth leading cause of blindness in 2020 globally. Thus, it is important to explore the pathogenesis and potential risk factors of DR.

Serum lipids are involved in the occurrence and progression of DR in T2DM; however, the results are controversial. The Chennai Urban Rural Epidemiology Study [7] showed that total cholesterol (TC), total triglyceride (TG), and low-density lipoprotein cholesterol (LDL-C) were associated with DR. After adjusting for glycosylated hemoglobin and the body mass index (BMI), only TG maintained a significant association with DR. Chen et al. reported TG as a risk factor for developing DR by multivariate Cox regression [8]. Furthermore, the poor controlling of TC was associated with the incidence of vision-threatening DR and macular edema, and higher TG levels were related to the progression to proliferative DR [9]. Benarous et al. [10] found that patients with higher LDL-C levels were more likely to have clinically significant macular edema when adjusting for age, gender, BMI, lipid-lowering agents, and some other factors. A multi-ethnicity-based cohort study suggested that higher LDL-C levels were risk factors for DR progression [11]. However, the evidence of the relationship between serum lipids and DR is inconsistent. The AusDiab study [12], which was the first national study on DR in a developed country, failed to show significant correlations between serum TC levels and TG levels and DR incidence. Additionally, Dai et al. reported no significant difference in baseline TC, TG, LDL-C, and high-density lipoprotein cholesterol (HDL-C) levels between patients with new-onset DR and those without DR [13]. A previous meta-analysis [14] showed a slightly higher level of LDL-C in the DR group than in the no-DR group; however, no significant differences were observed in TG, TC, and HDL-C levels between the two groups. In this meta-analysis published in 2018, seven studies with 4366 participants were included, among which 3879 (88.85%) had T2DM. Furthermore, they failed to differentiate the effects of serum lipids on patients with T1DM and T2DM. To date, no meta-analysis focusing exclusively on T2DM has been published.

In view of this, clarifying whether serum lipid levels affect the occurrence of DR in patients with T2DM is necessary. Therefore, we conducted a systematic review and meta-analysis to elucidate the preliminary relationship between serum lipid levels and DR incidence in patients with T2DM.

## Materials and methods

This study was registered with PROSPERO (registration number: CRD42022319978). This systematic review was conducted in accordance with the Preferred Reporting Items for Systematic Reviews and Meta-Analyses (PRISMA) guidelines [15].

### Search strategy and selection criteria

Published studies focusing on the relationship between serum lipids and DR in T2DM were identified through PubMed, Embase, and the Cochrane Library (until February 7, 2022). The terms or keywords used were as follows: (1) low-density lipoprotein OR high-density lipoprotein cholesterol OR triglyceride OR total cholesterol, (2) serum lipids OR dyslipidemia OR lipemia OR blood lipid profile, and (3) diabetic retinopathy. The reference lists of the included studies were also reviewed in order to find potentially relevant records. No language restriction was imposed. The detailed search strategy is shown in Additional file 4: Table S1.

### Inclusion and exclusion criteria

After removing duplicate studies, two investigators (QQJ and WQ) independently reviewed the titles, abstracts, and full texts to assess the eligibility of the studies. Any discrepancy was solved by discussion or consultation of another investigator (FL). The following are the inclusion criteria: (1) being cohort studies, (2) assessing the effect of the lipid level on DR incidence, and (3) the mean value and corresponding standard deviations (SDs) of blood lipid levels could be obtained. The exclusion criteria were as follows: (1) irrelevant articles, (2) cross-sectional or case–control studies or some other publication types (e.g., letters, comments, case reports, or reviews), and (3) inaccessibility of full-text.

### Data extraction

Data extraction was performed by two researchers independently. First author, year of publication, study design, sample size, country of origin, duration of follow-up, outcomes, mean values, and SDs of serum lipid concentrations were extracted. Any disagreements in the data extraction were resolved through discussion or consultation of another investigator (FL). All serum lipid data were expressed in mg/dL. To convert TC, LDL-C, and HDL-C to mg/dL, multiply by 38.66; to convert TG to mg/dL, multiply by 88.6.

### Quality assessment

We used the Newcastle–Ottawa Scale (NOS) to assess the methodological quality of the included studies in this meta-analysis. In NOS, the selection of cohorts, the comparability of cohorts, and the ascertainment of the exposure and outcome of interest were taken into account [16]. A higher score indicates a higher quality. Studies achieved score 6 or more are considered to be of high quality. The maximum score is nine.

### Statistical analysis

STATA 12.0 (StataCorp, College Station, TX, USA) was used for statistical analyses. The weighted mean differences (WMDs) and relevant 95 percent confidence intervals (CIs) (95% CIs) of TC, TG, LDL-C, and HDL-C levels between DR cases and the controls were pooled. Cochrane’s *Q* test and the *I*^2^ statistic were used to test for heterogeneity. The fixed effects model was applied to combine the summary estimates if heterogeneity index *I*^2^ was < 50%; otherwise, the random effects model was used [17]. A sensitivity analysis was performed by removing each study to identify whether the results could be affected markedly by a single study. Subgroup analysis was performed according to the country of origin and duration of follow-up. Potential publication bias was assessed by Begg’s test and Egger’s test [18]. Two-tailed *p*-value of < 0.05 was considered statistically significant.

## Results

### Characteristics of the studies and risk of bias

**Fig. 1:**
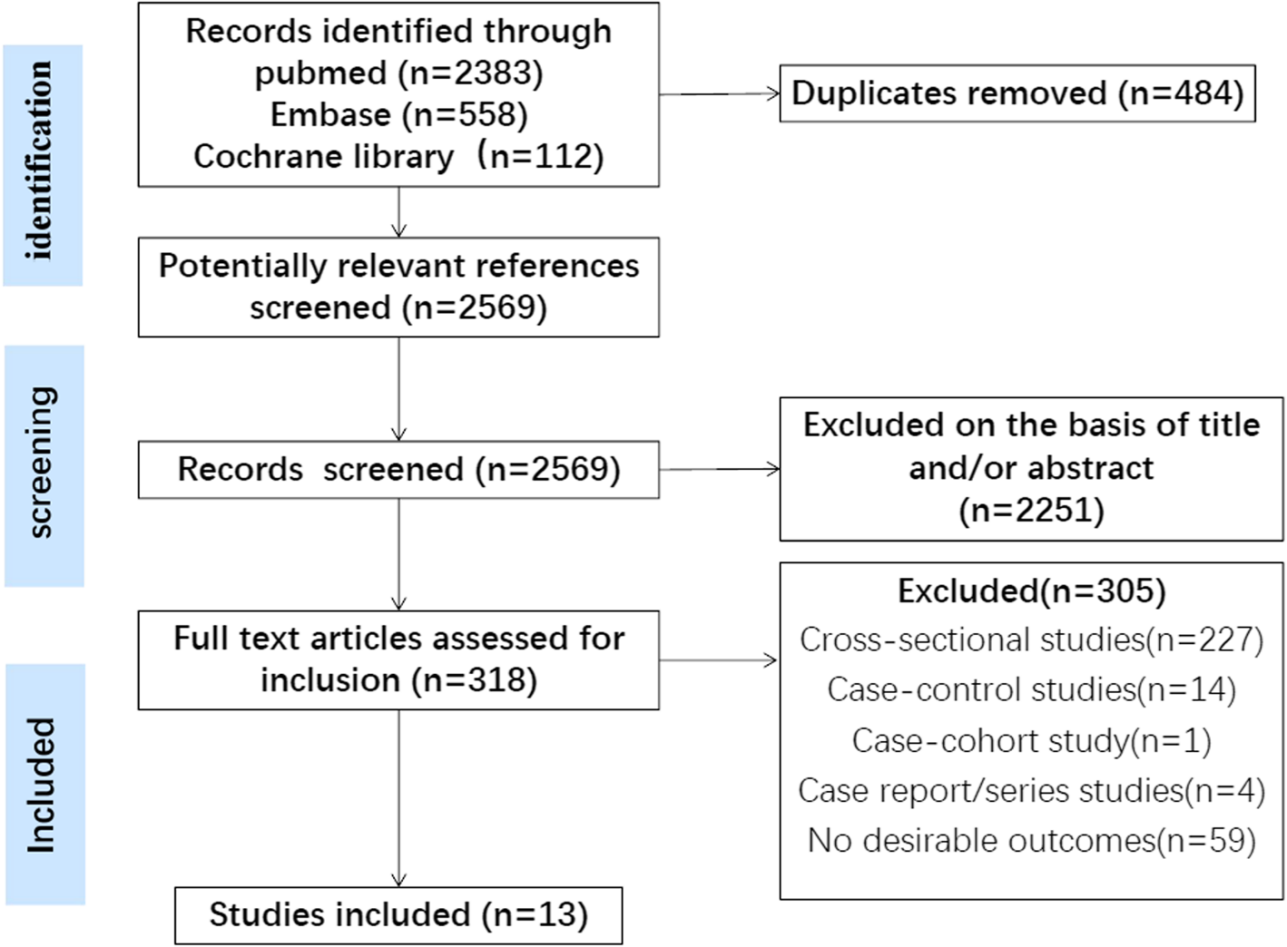
Flow diagram of the literature search and study selection

The PRISMA flow diagram of the study selection process in the present meta-analysis is presented in Fig. 1. Through the initial search, a total of 2569 articles were found, and of which 2251 records were excluded after screening their titles and the abstracts. By reviewing the full text of the remaining 318 articles, 305 articles were excluded for various reasons. Finally, 13 cohort studies [8, 11, 13, 19–28] including 7459 participants were considered relevant and included in the meta-analysis. The characteristics of the included studies are summarized in Table 1. Overall, the quality of the included studies was generally high or moderate, with ten studies scoring 7 and three studies scoring 6 points. The overall risk of bias was low to moderate (Additional file 1: Fig. S1). The details of assessing items for each study are available in Additional file 5: Table S2.

**Table 1:** Characteristics of the included studies

Author	Year	Regions	Age (years)	Male (*n* (%))	Duration of T2DM (years)	DR/NDR (*n*)	Exposures	Duration of follow-up (years)	NOS score
Chiu [19]	2021	Taiwan	67.85 (6.53)	339 (42.8%)	NR	611/181	TC, TG, HDL-C, LDL-C	NR	4
Cheung [11]	2021	USA	63.2 (8.9)	251 (50.4%)	9.4 (7.9)	70/295	TC, HDL-C, LDL-C	8	8
Chen [8]	2021	China	58.60 (10.55)	163 (49.4%)	7.09 (5.36)	30/300	TC, LDL-C	3.66 (1.90)	6
Dai [13]	2021	China	58.0 (10.1)	191 (60.6%)	10.7 (6.43)	81/234	TC, TG, HDL-C, LDL-C	3.42	6
Zhang [20]	2018	China	57.0 (5.8)	635 (62.1%)	4.81 (2.01)	315/708	TC, TG, HDL-C	10	6
Yun [21]	2016	South Korea	54.2 (10.0)	236 (42.4%)	6.3 (5.3)	263/293	TC, TG, HDL-C, LDL-C	11.1	7
Tseng [22]	2015	Taiwan	58.2 (13.1)	354 (52.6%)	10.68 (6.46)	91/482	TC, TG, HDL-C, LDL-C	2.9	5
Salinero-Fort [23]	2013	Spain	67.5 (10.6)	1217 (50.6%)	7.6 (7.2)	194/2211	TC, TG, HDL-C, LDL-C	4	8
Manaviat [24]	2008	Iran	55.2 (9.64)	31 (25.8%)	11.6 (6.2)	57/63	TC, TG	4	5
Tung [25]	2005	Taiwan	61.7 (11.6)	238 (43.4%)	7.59 (2.6)	93/455	TC,TG	2.56 (0.73)	7
Van Leiden [26]	2003	Netherlands	62.1 (6.8)	124 (53.2%)	NR	27/206	TC, TG, HDL-C	9.4	7
Tudor [27]	1998	USA	57.3 (9.7)	73 (43.2%)	4.52 (6.0)	47/122	TC, TG, HDL-C	4.8 (2.0–6.6)	8
Jarrett [28]	1986	UK	44–73	30 (100%)	NR	8/22	TC, TG	10	5

*NG* Not reported, *DR* Diabetic retinopathy, *NDR* No diabetic retinopathy, *NOS* Newcastle–Ottawa Scale, *TC* Total cholesterol, *TG* Total triglyceride, *LDL-C* Low-density lipoprotein cholesterol, *HDL-C* high-density lipoprotein cholesterol

### Meta-analysis results

**Fig. 2:**
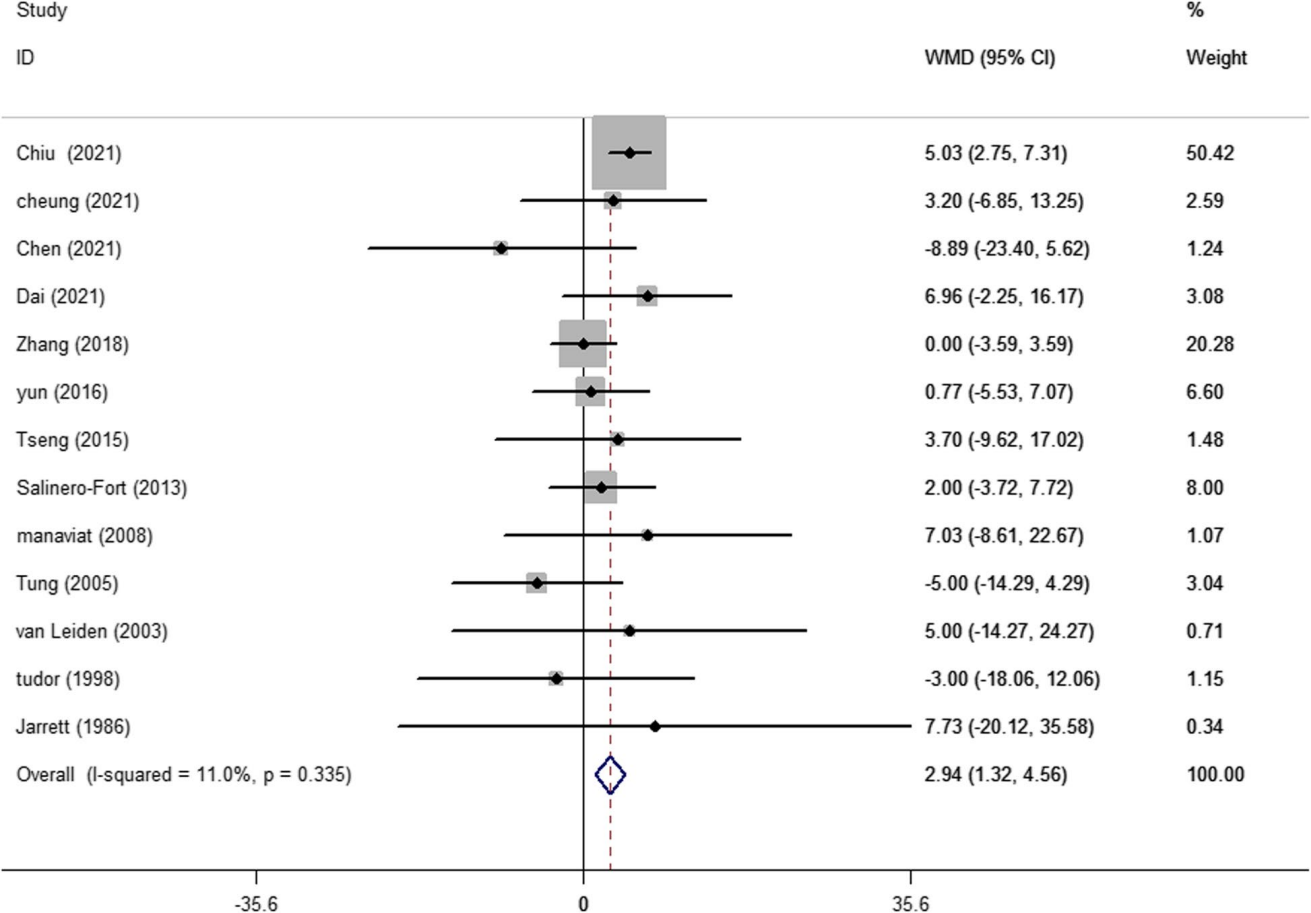
Forest plot of comparing TC levels between the DR population and the control group. WMDs, weighted mean differences; 95% CIs, 95% confidence interval; TC, total cholesterol; DR, diabetic retinopathy

**Fig. 3:**
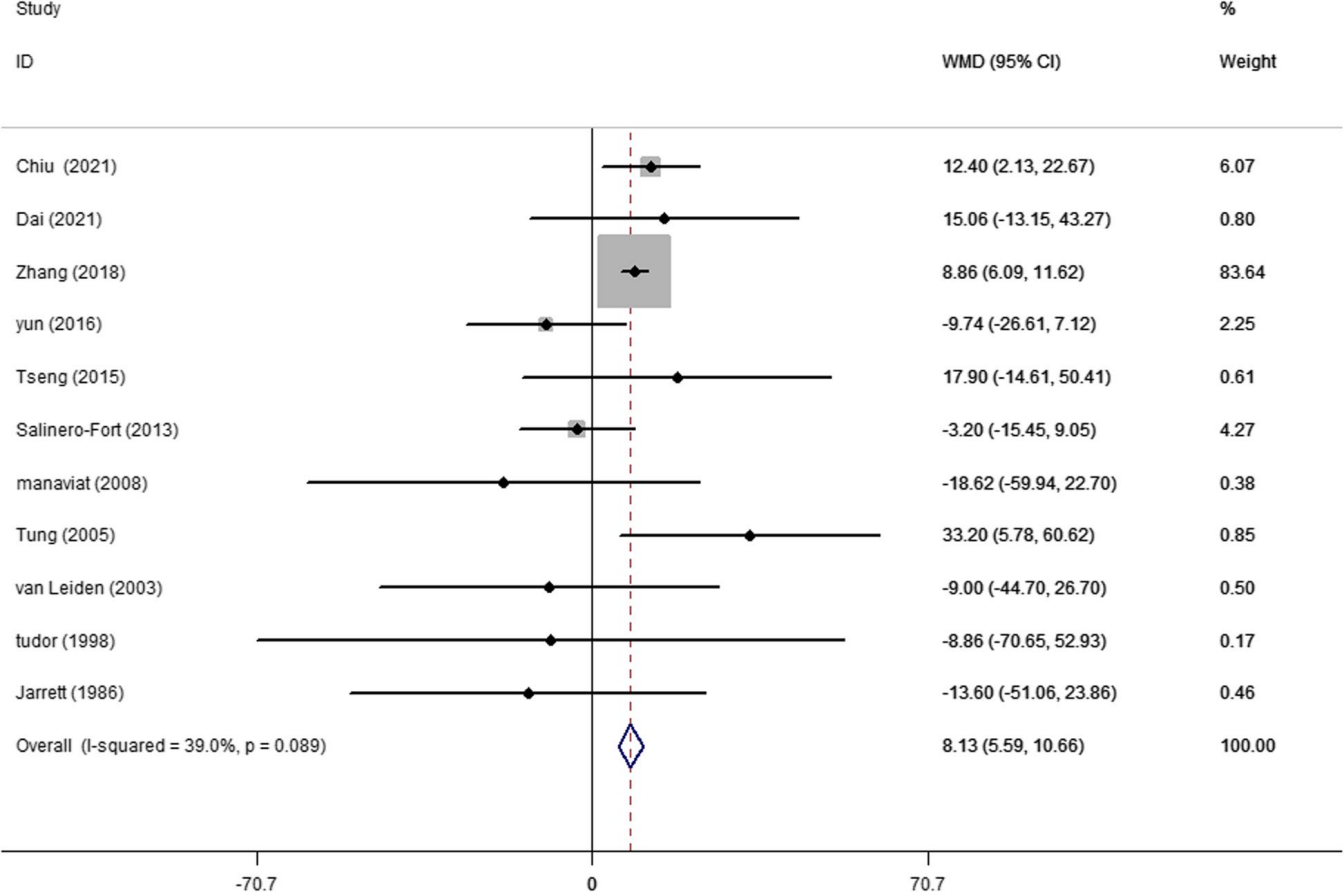
Forest plot of comparing TG levels between the DR population and the control group. WMDs, weighted mean differences; 95% CIs, 95% confidence interval; TG, total triglyceride; DR, diabetic retinopathy

**Fig. 4:**
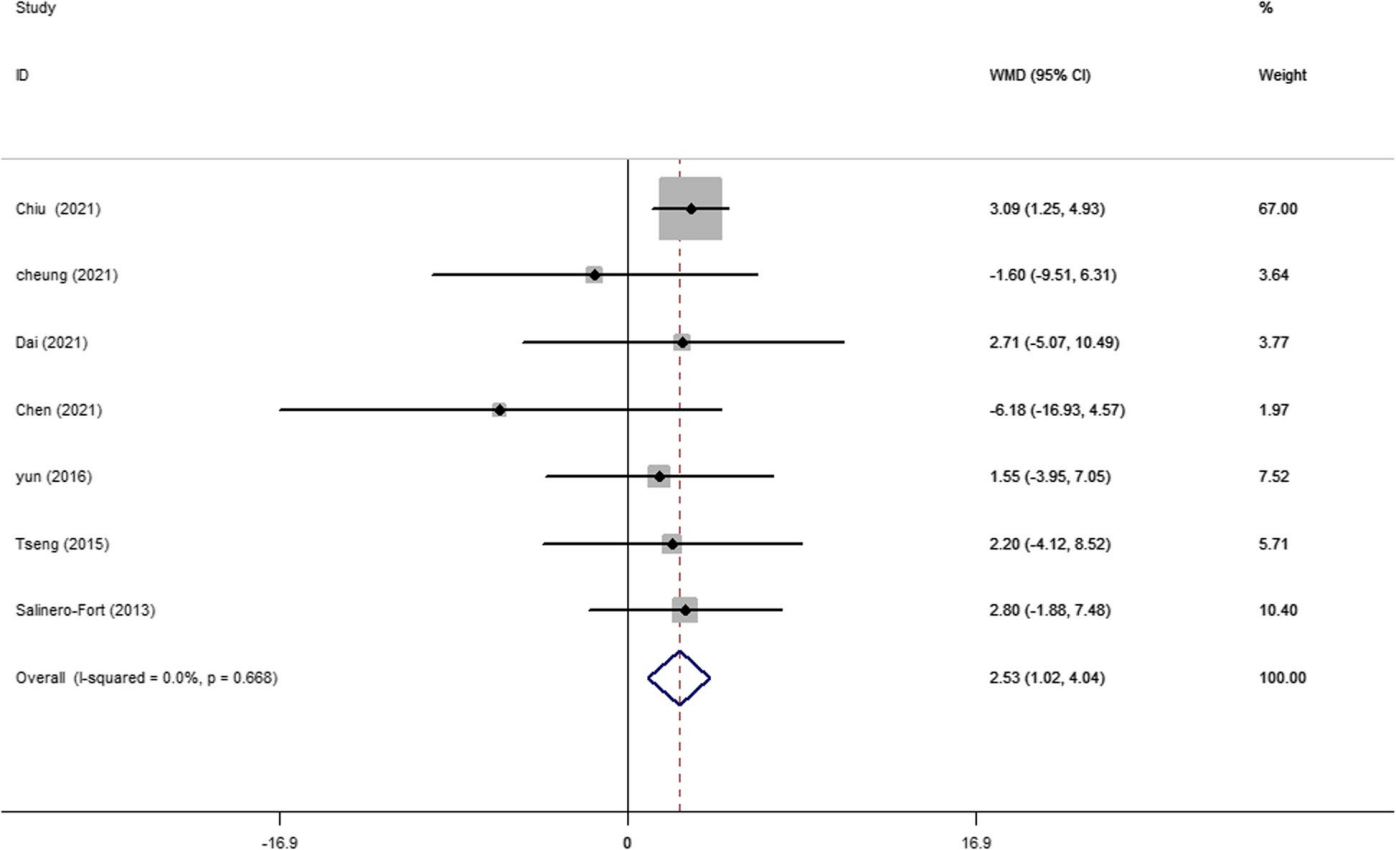
Forest plot of comparing LDL-C levels between the DR population and the control group. WMDs, weighted mean differences; 95% CIs, 95% confidence interval; LDL-C, low-density lipoprotein cholesterol; DR, diabetic retinopathy

**Fig. 5:**
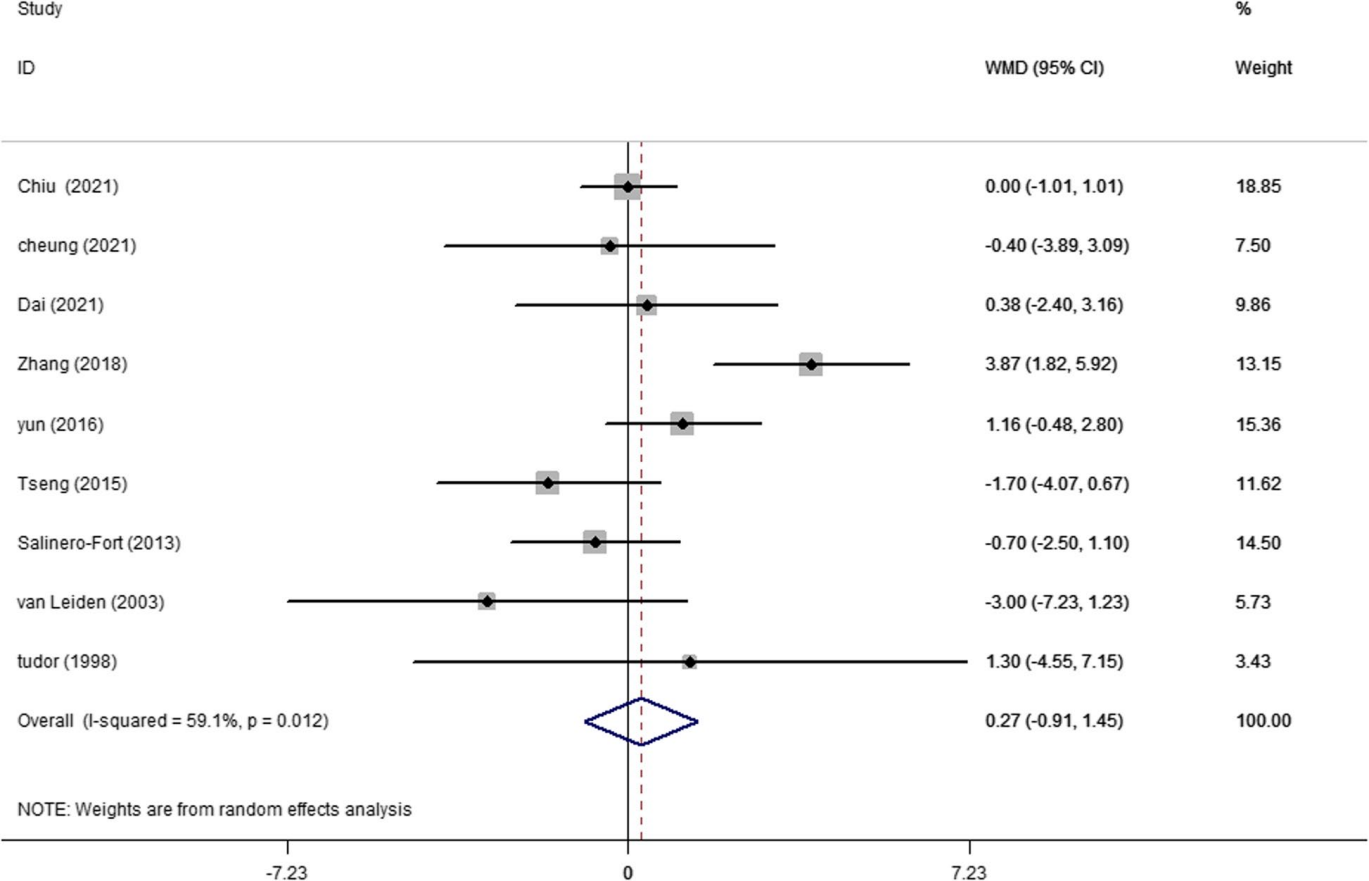
Forest plot of comparing HDL-C levels between the DR population and the control group. WMDs, weighted mean differences; 95% CIs, 95% confidence interval; HDL-C, high-density lipoprotein cholesterol; DR, diabetic retinopathy

A total of 13 articles evaluated baseline TC levels in patients with and without DR in T2DM. The pooling of these results revealed a significantly higher TC level (WMD 2.94 mg/dL, 95% CI 1.32, 4.56, *p* < 0.001) at baseline in participants who developed DR later on, with a fixed effects model (*I*^2^ = 11.0%, *p* heterogeneity = 0.335) (Fig. 2). A significantly higher baseline TG level (WMD 8.13 mg/dL, 95% CI 5.59, 10.66, *p* < 0.001; *I*^2^ = 39.0%, *p* heterogeneity = 0.089) was observed in cases who developed DR later on (Fig. 3). In addition, as shown in Fig. 4, the combined WMD of LDL-C suggested that LDL-C was significantly higher in DR cases (WMD 2.53 mg/dL, 95% CI 1.02, 4.04, *p* = 0.001) with a fixed effects model (*I*^2^ = 0%, *p* heterogeneity = 0.668). However, the present meta-analysis showed no significant difference in HDL-C levels (WMD 0.27 mg/dL, 95% CI − 0.91, 1.45, *p* = 0.656) between these two groups with a random effects model (*I*^2^ = 59.1%, *p* heterogeneity = 0.012) (Fig. 5).

### Exploration of the source of heterogeneity

To explore the source of heterogeneity, a sensitivity analysis was performed for HDL-C, for which *I*^2^ was 59.1%. After removing one influential study [20], the pooled result did not change significantly (WMD − 0.01 mg/dL, 95%CI: − 0.78, 0.59, *p* = 0.784) with a fixed effects model (*I*^2^ = 0%, *p* heterogeneity = 0.467). Because only nine studies on HDL-C were available, we did not conduct a meta-regression analysis as recommended by the Cochrane Handbook version 5.1.0 [29]. In the present study, the subgroup analysis was performed on the basis of the country of origin and duration of follow-up. The heterogeneity in the Asian group was high (*I*^2^ = 62.7%, *p* heterogeneity = 0.030), whereas no heterogeneity was observed in the “other” group (*I*^2^ = 0%, *p* heterogeneity = 0.721) (Additional file 2: Fig. S2A). Neither of the two subgroups (Asian vs. others) showed that HDL-C affected the occurrence of DR. When the studies were stratified on the basis of whether the follow-up time was over 4 years, no heterogeneity was observed in the group with a short follow-up time (*I*^2^ = 0%, *p* heterogeneity = 0.735); however, the heterogeneity in the “other” group was relatively high (*I*^2^ = 47.3%, *p* heterogeneity = 0.108) (Additional file 2: Fig. S2B). Similarly, the two subgroups showed no significant difference in HDL-C levels in participants who developed DR.

However, the duration of follow-up was not specified in the study by Chiu et al. [19]; thus, we conducted the meta-analysis by excluding this study. As shown in Additional file 3: Fig. S3A, no significantly higher TC level was observed in DR cases (WMD 0.81 mg/dL, 95% CI − 1.49, 3.11, *p* = 0.489) in the fixed effects model (*I*^2^ = 0.0%, *p* heterogeneity = 0.802). TG levels were significantly higher in patients with DR than in patients without DR (WMD 7.85 mg/dL, 95% CI 5.24, 10.46, *p* < 0.001) in the fixed effects model (*I*^2^ = 42.6%, *p* heterogeneity = 0.074) (Additional file 3: Fig. S3B). No significantly higher LDL-C levels were observed in DR cases (WMD 1.38 mg/dL, 95% CI − 1.25, 4.01, *p* = 0.303) in the fixed effects model (*I*^2^ = 0.0%, *p* heterogeneity = 0.704) (Additional file 3: Fig. S3C). Similarly, the pooled result showed no significant difference in HDL-C levels (WMD 0.032 mg/dL, 95% CI − 0.082, 0.146, *p* = 0.582) in the random effects model (*I*^2^ = 56%, *p* heterogeneity = 0.026) (Additional file 3: Fig. S3D).

### Publication bias

**Fig. 6:**
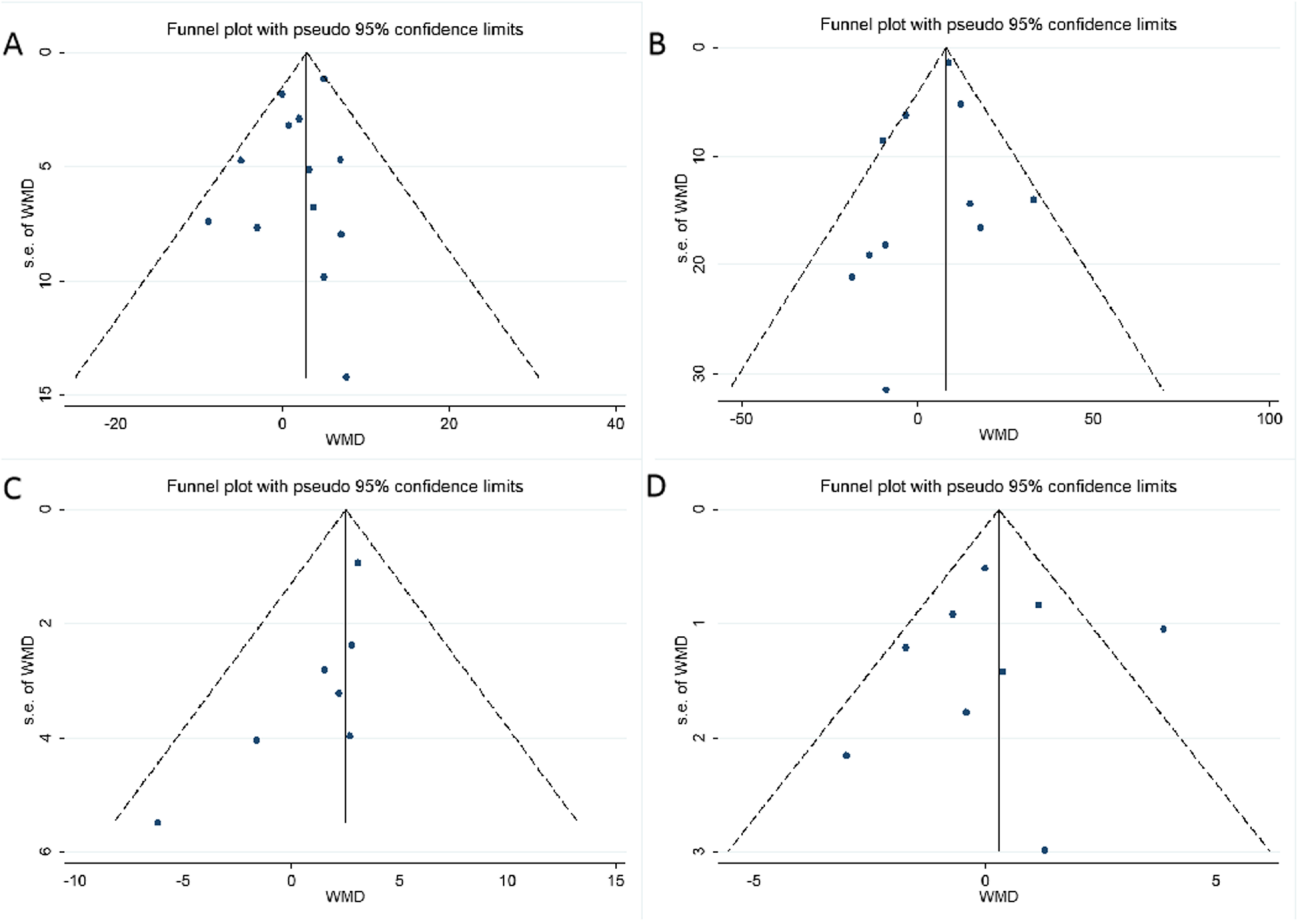
Funnel plots showing the risk of publication bias in the meta-analysis. **A** Funnel plot of TC. **B** Funnel plot of TG. **C** Funnel plot of LDL-C. **D** Funnel plot of HDL-C. WMDs, weighted mean differences; se, standard error; TC, total cholesterol; TG, total triglyceride; LDL-C, low-density lipoprotein cholesterol; HDL-C, high-density lipoprotein cholesterol

No evidence of publication bias was detected by Begg’s test and Egger’s test in TC (Begg’s test *p* = 0.502; Egger’s test *p* = 0.269), TG (Begg’s test *p* = 0.276, Egger’s test *p* = 0.313), and HDL-C (Begg’s test *p* = 0.917, Egger’s test *p* = 0.852). However, Begg’s and Egger’s tests suggested potential publication bias in LDL-C among the included studies (Begg’s test *p* = 0.035; Egger’s test *p* = 0.048). Visual inspection of the funnel plots did not lead to concerns about publication bias in TC, TG, and HDL-C but indicated little evidence of publication bias in LDL-C (Fig. 6). Thus, the results of LDL-C should be interpreted cautiously.

## Discussion

To the best of our knowledge, this is the first meta-analysis to evaluate the relationship between serum TC, TG, LDL-C, and HDL-C levels and DR incidence focusing on T2DM. The overall pooled results showed significantly higher lipid levels including TC, TG, and LDL-C in patients with later onset of DR than that in patients without DR. However, in this study, no significant difference in HDL-C levels were observed between the DR and control groups. Generally, our results suggest that lowering serum lipids may benefit patients with T2DM for preventing the DR incidence.

The present results are consistent with previous findings. Dyslipidemia probably was a risk factor for DR in Thai patients with T2DM [30]. In a hospital-based retrospective cohort study, Takele et al. [31] found that TC > 200 mg/dL was a significant predictor of DR with an adjusted hazard ratio of 2.22 (1.08–4.55). In a large community prospective cohort study, Jin et al. found that low TG level was an independently protective factor for the regression of DR in T2DM [32]. Another cohort study showed that LDL-C level was an important factor for the development of DR in older populations with T2DM in Taiwan [19].

A retrospective cohort study further supported the conclusion that patients with DR usually had a higher baseline TC, TG, and LDL-C levels than people without DR who were newly diagnosed with diabetes [33]. However, the present results are somewhat different from those of a previously published meta-analysis on diabetes [14], in which slightly higher LDL-C levels were observed, and the authors failed to discriminate significant differences in TC, TG, and HDL-C levels between the DR and control groups. Due to the limited number of studies included, they failed to make a subgroup analysis by diabetes types (T1DM vs. T2DM), although the effects of serum lipids on these two groups were different.

In the present study, we do not find HDL-C was a potentially related factor for the occurrence of DR in patients with T2DM. This result is different from the result of a previous study. According to the NO BLIND study [34], a multi-center and cross-sectional study, DR was independently associated with HDL-C levels (OR: 1.042, 95% CI 1.012–1.109) in patients with T2DM in Italy. Different study designs and patient populations may explain these discrepant findings.

In fact, factors that affect the diagnosis of DR and the determination of serum lipids may affect the results of the present meta-analysis. The physiological status and food consumption before blood sample collection can directly affect serum lipid levels. Nordestgaard et al. reported [35] that HDL-C levels were hardly affected by diet, while TG and LDL-C were significantly affected by diet. Alcohol consumption is known to be the leading cause of hyper-triacylglycerolemia [36, 37]. Moreover, strenuous exercise and body position may affect the blood lipid levels by affecting the fluid distribution in the blood vessels [38]. Another critical point was that the longitudinal changes in blood lipids could not be obtained in the present study. Additionally, the technology of the fundus camera, whether mydriasis or not, may affect the diagnosis of DR [39].

The heterogeneity in HDL-C levels between the included studies in the present study cannot be ignored (*I*^2^ = 59.1%). The included studies were conducted in different regions, ages, and durations of follow-up. In order to minimize the effect of heterogeneity, the random effects model was used when pooling HDL-C level results. Furthermore, a sensitivity analysis was conducted by omitting one study in each turn, and the pooled results were still stable. However, because the number of studies on HDL-C levels was insufficient for meta-regression analysis, we did not further explore it. Potential confounding factors including BMI, physical activity, and smoking, were not considered in this study.

The mechanisms linking blood lipid levels and DR were reported in previous studies. The upregulation of circulatory cytokines (e.g., VEGF-A, VEGF-D, and PlGF) resulting from dyslipidemia might be linked to the occurrence and development of DR [40]. Oxidative stress and endoplasmic reticulum stress were also associated with the development of DR [41]. Yang et al. [42] found that the mitochondrial damage induced by dyslipidemia might accelerate the apoptosis of retinal neurons, thus contributing to microvascular damage and retinal destruction in diabetes. Thus, maintaining serum lipid levels in a normal range is necessary for preventing DR occurrence. Moreover, the use of fenofibrate was beneficial for patients with DR. Fenofibrate stimulated the upregulation of CD34 or CD133 on hamatopoietic stem cells in patients with DR and thus might delay its progression [43].

Our study has some strengths. As far as we know, this is the first study examining the relationship between baseline serum lipid levels and DR incidence exclusively in patients with T2DM and also updates the findings of the previous meta-analysis. The results herein suggest significantly increased TC, TG, and LDL-C levels in DR cases, emphasizing the importance of maintaining normal serum lipids for T2DM. However, certain limitations of this study cannot be ignored. Firstly, because of the limited number of the included studies, DR stages were not differentiated. Secondly, the included studies covered diverse areas, but ethnicity, economic level, medical care, and some other potential influencing factors including BMI, physical activity, and smoking were not considered. Thirdly, the studies included were designed to analyze the relationship between baseline lipid levels and DR, and longitudinal changes in blood lipid levels were ignored. Finally, our study was limited by the possibility of publication bias.

In summary, higher baseline TC, TG, and LDL-C levels were found in patients with later onset of DR than that in patients without DR. However, the conclusion should be interpreted with caution because of publication bias and unknown confounders. It might be worthwhile to lower serum lipids in patients with T2DM. Future studies exploring the relationship between longitudinal changes in serum lipid levels and DR occurrence are warranted. The mechanisms underlying those effects also need systematic exploration.

### Supplementary Information

**Additional file 1: Fig. S1.** The overall risk of bias of the included studies.

**Additional file 2: Fig. S2.** Forest plot of comparing HDL-C levels between DR population and the control group with subgroup analysis. (A) by country of origin; (B) by the duration of follow-up. WMDs, weighted mean differences; 95% CIs, 95% confidence interval; HDL-C, high-density lipoprotein cholesterol; DR, diabetic retinopathy.

**Additional file 3: Fig. S3.** Forest plot of comparing serum lipid levels between DR population and the control group by removing one study. (A) TC; (B) TG; (C) LDL-C; (D) HDL-C. WMDs, weighted mean differences; 95% CIs, 95% confidence interval; TC, total cholesterol; TG, total triglyceride; LDL-C, low-density lipoprotein cholesterol; HDL-C, high-density lipoprotein cholesterol; DR, diabetic retinopathy.

**Additional file 4: Table S1.** Search strategy of this meta-analysis.

**Additional file 5: Table S2.** The methodological quality of cohort studies in accordance with the Newcastle-Ottawa Scale (NOS).

**Additional file 6. **PRISMA 2020 Checklist.

## Data Availability

The data sets used for all analyses can be obtained from the corresponding author upon reasonable request.
