# Supplementary figures and images for: Relationship between dyslipidemia and diabetic retinopathy in patients with type 2 diabetes mellitus: a systematic review and meta-analysis
Source: Syst Rev. 2023 Aug 24;12:148. doi: 10.1186/s13643-023-02321-2 (PMC10463379; doi:10.1186/s13643-023-02321-2)

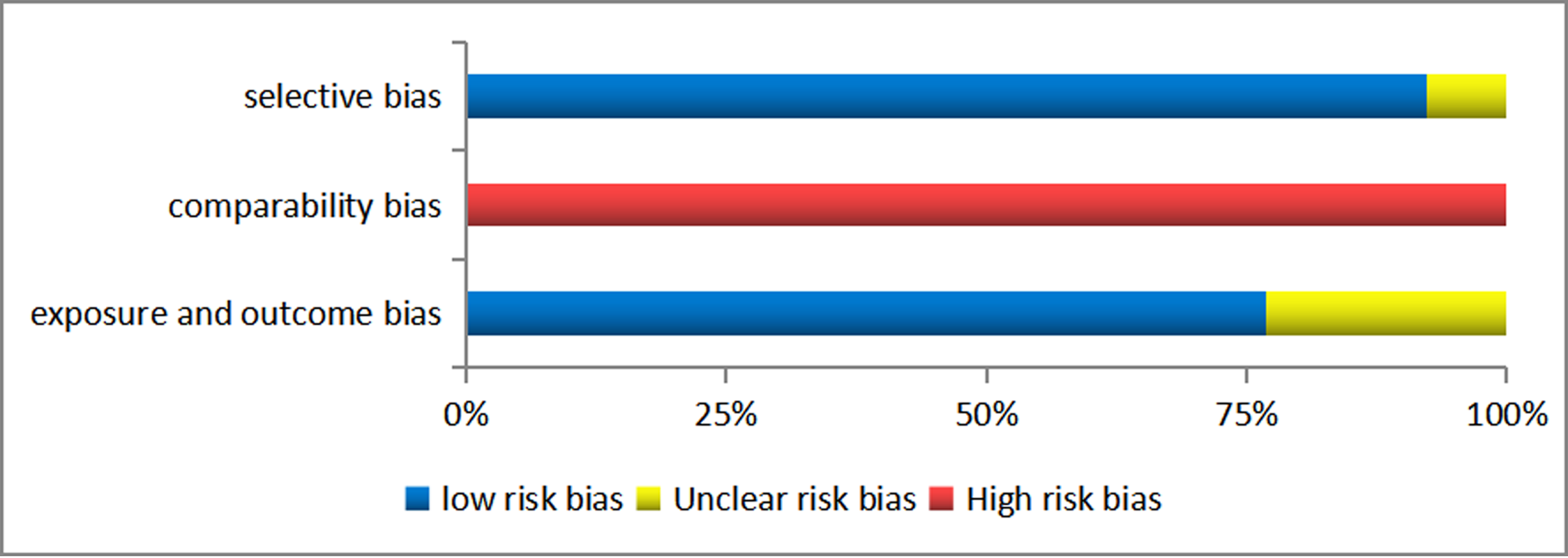

Supplement: Supplementary file 1 — Additional file 1: Fig. S1. The overall risk of bias of the included studies. [file 13643_2023_2321_MOESM1_ESM.tif]

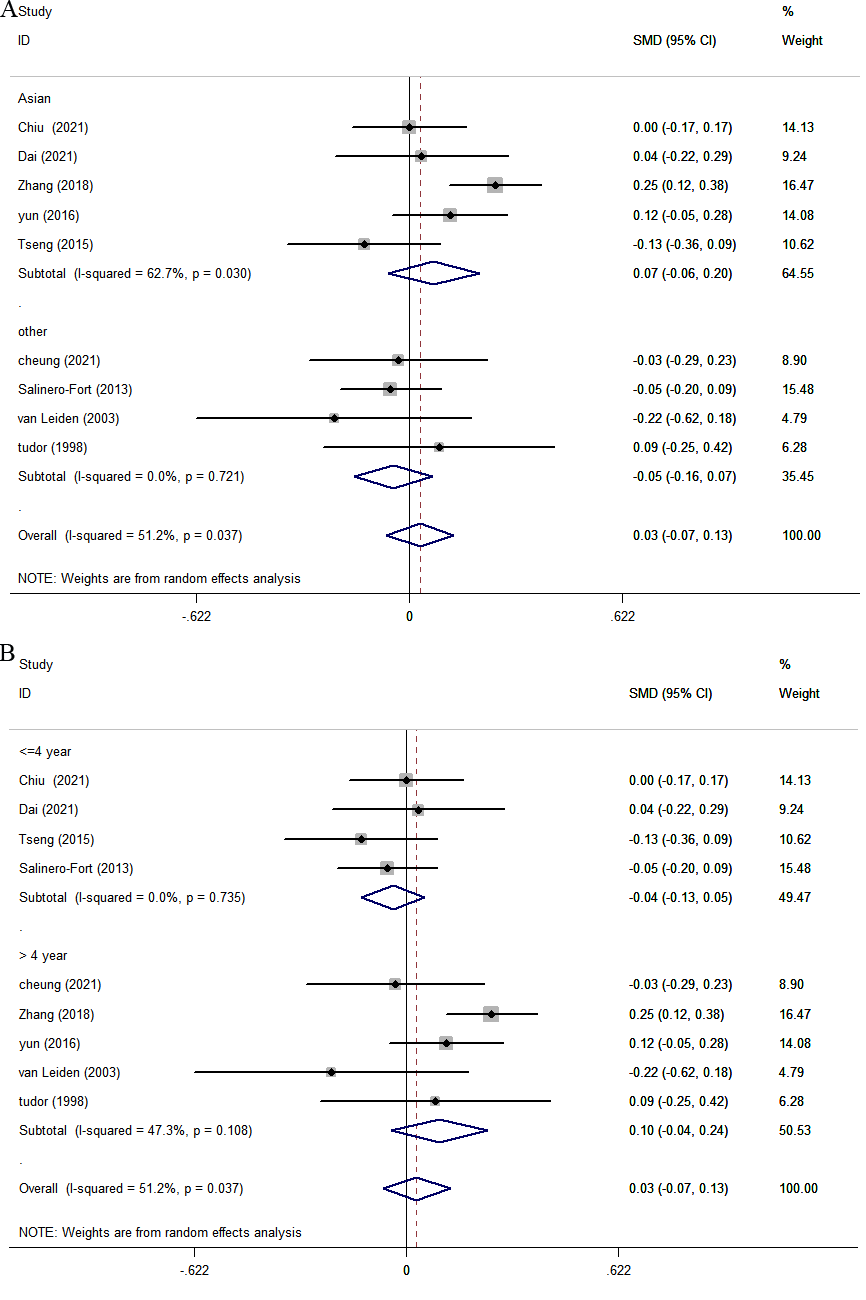

Supplement: Supplementary file 2 — Additional file 2: Fig. S2. Forest plot of comparing HDL-C levels between DR population and the control group with subgroup analysis. (A) by country of origin; (B) by the duration of follow-up. WMDs, weighted mean differences; 95% CIs, 95% confidence interval; HDL-C, high-density lipoprotein cholesterol; DR, diabetic retinopathy. [file 13643_2023_2321_MOESM2_ESM.tif]

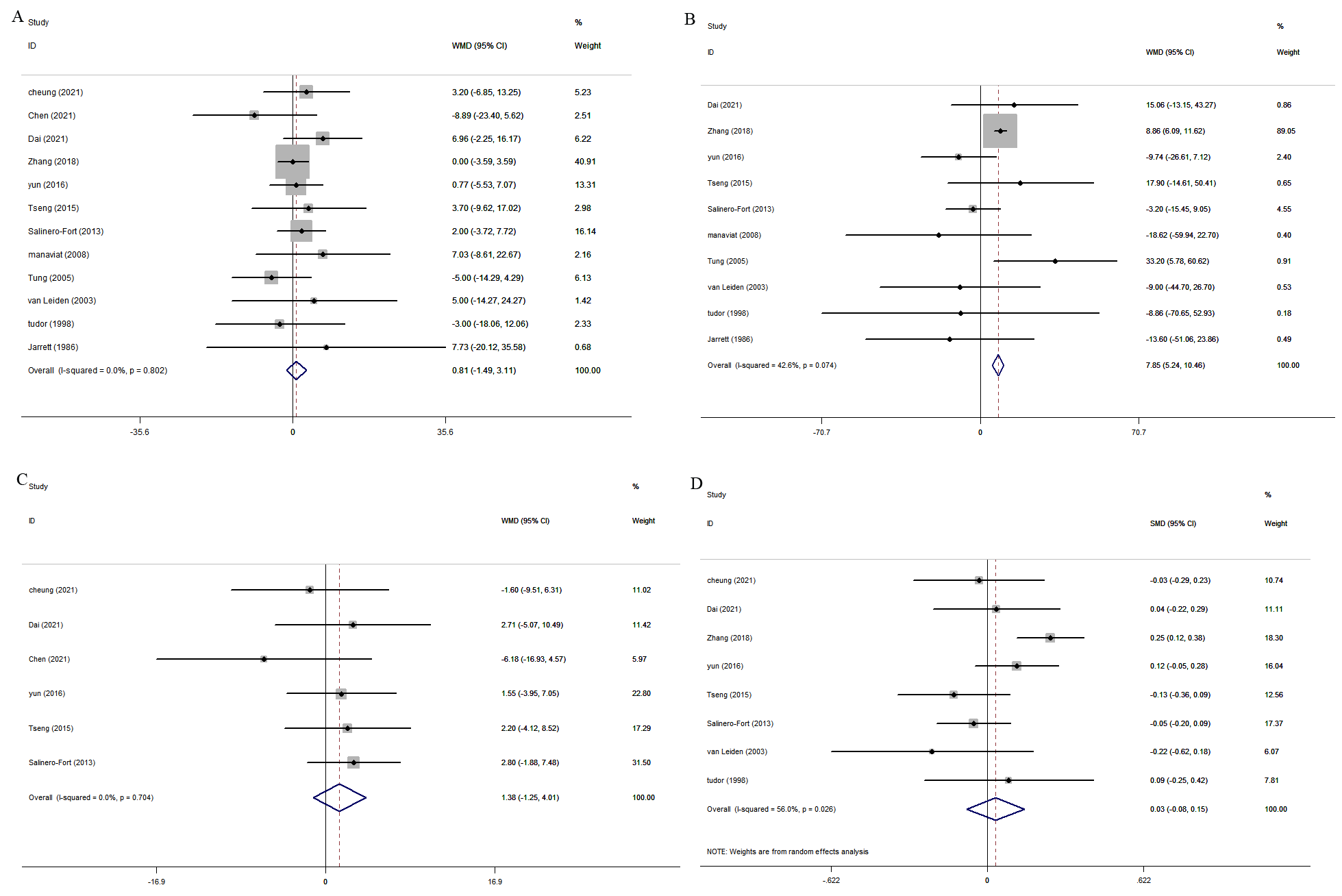

Supplement: Supplementary file 3 — Additional file 3: Fig. S3. Forest plot of comparing serum lipid levels between DR population and the control group by removing one study. (A) TC; (B) TG; (C) LDL-C; (D) HDL-C. WMDs, weighted mean differences; 95% CIs, 95% confidence interval; TC, total cholesterol; TG, total triglyceride; LDL-C, low-density lipoprotein cholesterol; HDL-C, high-density lipoprotein cholesterol; DR, diabetic retinopathy. [file 13643_2023_2321_MOESM3_ESM.tif]
