# Supplementary material for: Relationship between dyslipidemia and diabetic retinopathy in patients with type 2 diabetes mellitus: a systematic review and meta-analysis
Source: Syst Rev. 2023 Aug 24;12:148. doi: 10.1186/s13643-023-02321-2 (PMC10463379; doi:10.1186/s13643-023-02321-2)
Supplement: Supplementary file 4 — Additional file 4: Table S1. Search strategy of this meta-analysis. [file 13643_2023_2321_MOESM4_ESM.docx]

**Supplementary Table 1 search strategy of this meta-analysis**

| Database | search strategy |
| --- | --- |
| pubmed | ((low density lipoprotein cholesterol) OR (high density lipoprotein cholesterol) OR (serum lipids) OR (dyslipidemia) OR (lipemia) OR (blood lipid profile) OR (triglyceride) OR (total cholesterol)）AND (diabetic retinopathy) 2383 |
| embase | #3 #1 AND #2 558 |
|  | #2 ‘diabetic retinopathy’/exp OR ‘diabetic retinopathy’ OR ((diabetic’/exp OR diabetic) AND (retinopathy’/exp OR retinopathy)) 67273 |
|  | #1 ‘low density lipoprotein cholesterol’/exp OR ‘low density lipoprotein cholesterol’ OR (low AND ‘density’exp OR density) AND (‘lipoprotein’/exp OR lipoprotein) AND (‘cholesterol’/exp OR cholesterol)) OR ‘high density lipoprotein cholesterol’/exp OR ‘high density lipoprotein cholesterol’ OR (‘high density’ AND ‘lipoprotein’/exp OR lipoprotein AND (‘cholesterol’/exp OR cholesterol)) OR ‘serum lipids’ OR ((‘serum’/exp OR ‘serum’) AND (‘lipids’/exp OR lipids)) OR ‘dyslipidemia’exp OR ‘dyslipidemia’ OR ‘triglyceride’/exp OR ‘triglyceride’ OR ‘total cholesterol’/exp OR ‘total cholesterol’ OR ((‘total’/exp OR ‘total’) AND (‘cholesterol’exp OR ‘cholesterol’))) 77380 |
| Cochrane library | #3 #1 AND #2 112 |
|  | #2 MeSH descriptor: [Diabetic Retinopathy] explode all trees 1938 |
|  | #1 (cholesterol):ti,ab,kw OR (serum lipids):ti,ab,kw OR (dyslipidemia):ti,ab,kw OR (lipoprotein):ti,ab,kw OR (triglyceride):ti,ab,kw (Word variations have been searched) 55267 |
