# Supplementary material for: Relationship between dyslipidemia and diabetic retinopathy in patients with type 2 diabetes mellitus: a systematic review and meta-analysis
Source: Syst Rev. 2023 Aug 24;12:148. doi: 10.1186/s13643-023-02321-2 (PMC10463379; doi:10.1186/s13643-023-02321-2)
Supplement: Supplementary file 5 — Additional file 5: Table S2. The methodological quality of cohort studies in accordance with the Newcastle-Ottawa Scale (NOS). [file 13643_2023_2321_MOESM5_ESM.docx]

| Study | Selection | | | |  | Comparability |  | Outcome | | | Total score |
| --- | --- | --- | --- | --- | --- | --- | --- | --- | --- | --- | --- |
|  | Representativeness of the exposed cohort | Selection of the non-exposed cohort | Ascertainment of exposure | Demonstration that outcome of interest was not present at start of study |  | Comparability of cohorts on the basis of the design or analysis |  | Assessment of outcome | Was follow-up long enough for outcomes to occur? | Adequacy of follow up of cohorts? |  |
| Cheung 2021 | 1 | 1 | 1 | 0 |  | 2 |  | 1 | 1 | 1 | 8 |
| Chen 2021 | 0 | 1 | 1 | 1 |  | 0 |  | 1 | 1 | 1 | 6 |
| Chiu 2021 | 0 | 0 | 1 | 0 |  | 0 |  | 1 | 1 | 1 | 4 |
| Dai 2021 | 0 | 0 | 1 | 1 |  | 1 |  | 1 | 1 | 1 | 6 |
| Zhang 2018 | 0 | 1 | 1 | 1 |  | 0 |  | 1 | 1 | 1 | 6 |
| Yun 2016 | 0 | 1 | 1 | 1 |  | 1 |  | 1 | 1 | 1 | 7 |
| Tseng 2015 | 0 | 1 | 1 | 1 |  | 0 |  | 1 | 1 | 0 | 5 |
| Salinero-Fort 2013 | 1 | 1 | 1 | 1 |  | 1 |  | 1 | 1 | 1 | 8 |
| Manaviat 2008 | 0 | 1 | 1 | 0 |  | 1 |  | 1 | 1 | 0 | 5 |
| Tung 2005 | 1 | 1 | 1 | 0 |  | 1 |  | 1 | 1 | 1 | 7 |
| Van Leiden 2003 | 1 | 1 | 1 | 0 |  | 1 |  | 1 | 1 | 1 | 7 |
| Tudor 1998 | 1 | 1 | 1 | 0 |  | 2 |  | 1 | 1 | 1 | 8 |
| Jarrett 1986 | 0 | 0 | 1 | 0 |  | 1 |  | 1 | 1 | 1 | 5 |

**Supplementary table 2 The methodological quality of cohort studies in accordance with the Newcastle-Ottawa Scale (NOS)**
